# The Role of Ki67 in Evaluating Neoadjuvant Endocrine Therapy of Hormone Receptor-Positive Breast Cancer

**DOI:** 10.3389/fendo.2021.687244

**Published:** 2021-11-03

**Authors:** Ailin Zhang, Xiaojing Wang, Chuifeng Fan, Xiaoyun Mao

**Affiliations:** ^1^ Department of Breast Surgery, The First Affiliated Hospital of China Medical University, Shenyang, China; ^2^ Department of Pathology, First Affiliated Hospital and College of Basic Medical Sciences of China Medical University, Shenyang, China

**Keywords:** neoadjuvant endocrine therapy, breast cancer, Ki67, hormone-positive breast cancer, clinical marker

## Abstract

Ki67 is a proliferation marker. It has been proposed as a useful clinical marker for breast cancer subtype classification, prognosis, and prediction of therapeutic response. But the questionable analytical validity of Ki67 prevents its widespread adoption of these measures for treatment decisions in breast cancer. Currently, Ki67 has been tested as a predictive marker for chemotherapy using clinical and pathological response as endpoints in neoadjuvant endocrine therapy. Ki67 can be used as a predictor to evaluate the recurrence-free survival rate of patients, or its change can be used to predict the preoperative “window of opportunity” in neoadjuvant endocrine therapy. In this review, we will elaborate on the role of Ki67 in neoadjuvant endocrine therapy in breast cancer.

## Introduction

Ki67 is a nuclear antigen that is an excellent marker of active cell proliferation in the normal and tumor cell populations (1). It has been proposed as a useful clinical marker for breast cancer subtype classification, prognosis, and prediction of therapeutic response (2–4). But the questionable analytical validity of Ki67 prevents its widespread adoption of these measures for treatment decisions in breast cancer (5). Previous study suggested that baseline Ki67 and its change after short-term endocrine treatment (e.g., 2 weeks) have predictive value of recurrence-free survival (6). Currently, several studies have investigated the possible use of Ki67 assessment in neoadjuvant endocrine therapy (NET). This review assessed the role of Ki67 in NET of breast cancer.

## Ki67 Structure and Biological Function

**Figure 1:**
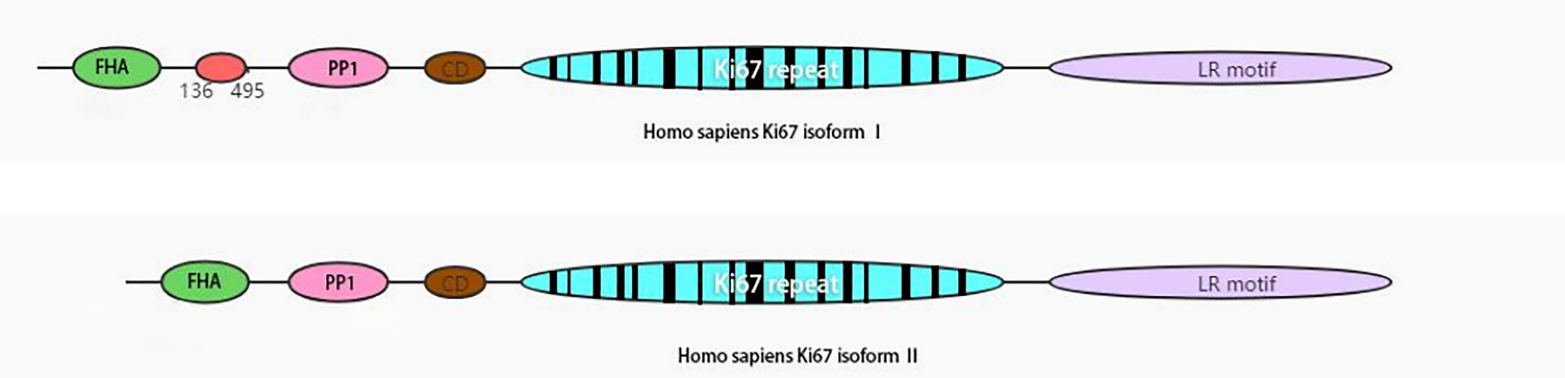
Schematic view of human Ki67 protein structure. The isoform II lacks amino acid 136-495. FHA, forkhead-associated domain; PP1, PP1-binding domain; CD, conserved domain; LR, leucine-arginine-rich domain.

Ki67 is expressed in all active phases of the cell cycle (late G1 phase and subsequent S, G2, and M phases), peaks in M phase, dissipates rapidly after mitosis, and is not expressed in stationary G0 phase (7). It is encoded by MKI67 and maps to human 10q26.2. It has a potential phosphorylation site for a range of essential kinases, PEST^1^ sequences, and a forkhead-associated domain (8) (**Figure 1**). It acts as an early protein to bind the perichromosomal layer in mitosis at the transition from prophase to prometaphase (9). During mitosis, Ki67 stabilizes and maintains the mitotic spindle and prevents chromosomes from collapsing into a single chromatin mass after nuclear envelope disassembly, thus enabling independent chromosome motility and efficient interactions with the mitotic spindle (10, 11). The tandem repeat group of Ki67 contains residues of Cyclin dependent kinase 1 (CDK1) phosphorylation during mitosis (12, 13), and many biological functions of Ki67 have subsequently been shown to be related to phosphorylation (8).

## Ki67 in Breast Cancer

Ki67 is a marker of cell proliferation. In normal healthy breast tissue, very low levels of Ki67 (<3%) have been reported (14, 15). Previous research indicated that estrogen receptor (ER)-positive cells did not proliferate in rodent mammary gland; ERα receives the proliferation signal from E2, initiates DNA synthesis, and is then lost from cells (16). The subsequent steps in proliferation can proceed in the absence of either ERα or ERβ (16). Ki67 is expressed exclusively in ER-negative cells in normal breast tissue (15, 17, 18). Ki67 expression is significantly higher in hyperplastic enlarged lobular units than in adjacent normal terminal duct lobular units (average 6.3% vs. 2.0%; P < 0.0001) (19) and is related to the subsequent risk of breast cancer (14, 20, 21). The exclusive Ki67 expression pattern with ER is disrupted during breast carcinogenesis (22, 23). Numerous studies have indicated that early-stage breast cancer patients with high Ki67 expression have a higher risk of recurrence and poorer survival rate (3, 24–27). The International Ki67 in Breast Cancer Working Group (IKWG) accepted that Ki67 immunohistochemistry (IHC) as a prognostic marker in breast cancer has limited clinical validity at present. Ki67 IHC is used as a prognostic marker in early breast cancer regarding whether further adjuvant chemotherapy is warranted to predict or monitor chemotherapy response (28). Ki67 IHC is a useful tool in assessing the risk of recurrence for ER-positive human epidermal growth factor receptor 2 (HER2)-negative breast cancers, where it may be considered a surrogate of molecular assays for distinguishing luminal A from luminal B breast cancer subtypes. High Ki67 has been reported to be associated with a good clinical response to chemotherapy (3), especially in triple-negative breast cancer (15, 29). But it had limited independent significance and does not merit measurements in most routine clinical scenarios. A clinical trial from the European Institute of Oncology indicated that high Ki67 (≥32%) can benefit from adjuvant chemotherapy in luminal B breast cancer with positive lymph node metastasis (30). Penault-Llorca et al. (31) reported that a high Ki67 index (≥20%) in the PACS01 trial was linked with a higher efficacy of docetaxel in adjuvant therapy for ER-positive breast cancer. The BCIRG001 clinical trial found that docetaxel, doxorubicin, and cyclophosphamide (TAC) chemotherapy regimen had a significant complementary effect on endocrine therapy for patients with a high Ki67 index (≥13%), ER positivity, and lymph node positivity (32). In IBCSG trials VIII and IX, high Ki67 index (≥19%) correlated with poor disease-free survival among 1,521 patients with endocrine-reactive breast cancer (33). Ki67 index is a valuable prognostic indicator in endocrine-responsive breast cancer without lymph node metastasis, but it is not a predictive factor of better response to adjuvant chemotherapy in these studies (30, 34).

## Ki67 in Neoadjuvant Endocrine Therapy

The efficacy evaluation of neoadjuvant chemotherapy (NAC) is mainly based on the clinical response and pathologic response tumor and lymph nodes after treatment (35). In NET, Ki67 has been tested as a predictive marker for chemotherapy using clinical and pathological responses as endpoints (36). Several large clinical trials of NET have assessed the change of Ki67 as an endpoint (37–39) (**Table 1**). Two important clinical trials of NETs, the Immediate Preoperative Anastrozole, Tamoxifen, or Combined with Tamoxifen (IMPACT) trial and P024, established Ki67 as the evaluation index of NETs. IMPACT compared the efficacy of NET with anastrozole, tamoxifen, and a combination of anastrozole and tamoxifen in postmenopausal women with ER-positive invasive primary breast cancer (45). P024 compared letrozole with tamoxifen in NET (40, 46). IMPACT is a clinical trial similar to the ATAC (Arimidex, Tamoxifen, Alone or in Combination) trial, which compared 5 years of the aromatase inhibitor anastrozole alone, tamoxifen alone, and their combination as adjuvant therapy in postmenopausal women with localized breast cancer. IMPACT was designed to test the hypothesis that the clinical response or the change in Ki67 predicts the outcome of ATAC (39). The ATAC trial is the largest adjuvant trial with the longest follow-up data to date, with 24,522 woman-years of follow-up in the anastrozole group and 23,950 woman-years in the tamoxifen group (47, 48). The results of this study are valuable, and its data continue to demonstrate improved efficacy for 5 years of anastrozole over tamoxifen alone. IMPACT has a similar design to ATAC in NET, avoiding a large number of patients and long follow-up time required for the efficacy evaluation of adjuvant trials, and aims to compare the recurrence and death risk of hormone receptor-positive patients in three NET regimens. The IMPACT trial required only 330 patients and a follow-up of just 3 months to provide its primary endpoint (40). In IMPACT, the change of Ki67 was greater in the anastrozole group than in the other groups at 2 weeks and 12 weeks, which closely parallels the results of the relative recurrence-free survival with adjuvant endocrine therapy after long follow-up in the ATAC trial in 9,366 patients. The short-term changes in Ki67, not the clinical evaluation (tumor size) in NET, might predict the long-term outcome during adjuvant use of the same treatments.

**Table 1:** Main neoadjuvant endocrine trials.

Clinical trials	Clinical response	Ki67 outcome
P024 (40–42)	ORR letrozole 55% vs. tamoxifen 36% (P < 0.001); ultrasound response letrozole 35% vs. tamoxifen 25% (P < 0.05); mammographic response letrozole 34% vs. tamoxifen 16% (P < 0.001); breast-conserving surgery letrozole 45% vs. tamoxifen 35% (P = 0.022).	No interaction with treatment-induced changes in Ki67 or absolute posttreatment Ki67 levels in either tamoxifen- or letrozole-treated tumor samples. Letrozole inhibited Ki67 to a greater extent than tamoxifen did (Ki67 geometric mean reduction 87% vs. 75%, respectively; P = 0.0009).
IMPACT (39)	There were no significant differences in OR in anastrozole, tamoxifen, or combination.	Greater Ki67 reduction in anastrozole arm. Ki67 geometric mean reduction: anastrozole 76% at 2 weeks/82% at 12 weeks; tamoxifen 59% at 2 weeks/62% at 12 weeks; combination 64% at 2 weeks/61% at 12 weeks.
ACOSOG Z1031 (43)	CRR letrozole 75% vs. exemestane 63% vs. anastrozole 69%.	No significance difference in Ki67 geometric mean reduction. Anastrozole 79% vs. exemestane 79% vs. letrozole 82%. Ki67-based data are closely equivalent with the data in adjuvant endocrine trials, therefore predicting similar activity as adjuvant therapies.
PROACT (44)	In hormonal therapy-only patients, ORR favored anastrozole arm (anastrozole 33% vs. tamoxifen 27%, P = 0.04), feasible surgery at baseline improved after 3 months in 43% of patients receiving anastrozole and 31% receiving tamoxifen (P = 0.04).	No data about Ki67

ORR, overall response rate; CRR, complete response rate.

P024 was a randomized, multinational, double-blind study comparing 4 months of letrozole vs. tamoxifen in postmenopausal women with hormone-responsive primary untreated breast cancer (41). P024 indicated that the percentage of Ki67-positive cells, pathological tumor size, lymph node status, and ER status were independently associated with breast cancer-specific survival and relapse-free survival. Based on these factors, Ellis et al. (46) obtained a clinically valuable prognostic model of preoperative endocrine prognostic index (PEPI) score for the outcome prediction of hormone-positive breast cancer with NET. The Ki67 and PEPI triage approaches can predict the risk of relapse. NET was initially an option for breast cancer patients who were too frail to have surgery or cytotoxic chemotherapy. It is very difficult to evaluate the efficacy of adjuvant endocrine therapy because of its long-term follow-up, and NET offers useful clues. The initial endocrine neoadjuvant therapy clinical trial collected data to evaluate the long-term outcome of adjuvant endocrine therapy indirectly rather than as a neoadjuvant treatment (49–51). Future adjuvant endocrine therapy clinical research designs should be based on a biological superiority hypothesis generated by a neoadjuvant endocrine study (52).

After almost 20 years of clinical studies on NETs, with considerable response rates in HR-positive breast cancer, NETs could be a significantly less toxic alternative to NAC for a subgroup of endocrine therapy-responsive breast cancer. The Z1031A trial enrolled postmenopausal women with large (stage II/III) ER-positive breast cancer with random anastrozole, exemestane, or letrozole NET. Subsequently, in Z1031B, the trial protocol was amended to include Ki67 determination after 2–4 weeks of neoadjuvant aromatase inhibitor therapy (53). If Ki67 was >10%, patients were switched to neoadjuvant chemotherapy on the basis of a presumptive lack of hormonal therapy benefit. A pathologic complete response (pCR) rate of >20% was the predefined efficacy threshold. With >5 years of median follow-up, only 3.7% (4/109) with a PEPI score of 0 relapsed vs. 14.4% (49/341) with a PEPI score >0. The Ki67 and PEPI algorithms can be used to evaluate relapse risk after NET. Miller et al. (54) collected 63 postmenopausal breast cancer patients with neoadjuvant letrozole for 3 months. Reduction in Ki67 >40% between pretreatment and 10–14 days was related to pathological responses. A pooled analysis of two multicenter, randomized, noncomparative, phase 2 clinical trials (HORGEN and CARMINA02) evaluating neoadjuvant anastrozole and fulvestrant efficacy for postmenopausal HR+/HER2- breast cancer indicated that PEPI can identify a subset of patients with poorer prognosis who should be offered all appropriate adjuvant therapy (55). Ki67 in neoadjuvant trials predicted the long-term outcomes of large adjuvant trials; Ki67 and PEPI can be predictors for evaluating the recurrence-free survival of breast cancer patients with NET (50). Early breast cancer patients with a PEPI = 0 have little to gain from adding additional adjuvant systemic therapy to their endocrine therapy (46).

The postmenopausal women with hormone-sensitive early breast cancer (POETIC) study was a phase 3 trial in which postmenopausal hormone receptor-positive early breast cancer patients were randomly assigned to POAI (letrozole or anastrozole) for 14 days before and following surgery or no POAI (control) (38). The data from POETIC showed that the patients with a low baseline Ki67 (<10%) had a low risk of recurrence (4.3% in HER-2-negative breast cancer, 10.1% in HER-2-positive breast cancer), and those with a high baseline Ki67 (≥10%) with conversion to low Ki67 after 2 weeks of NET had a high recurrence (21.5% in HER-2-negative breast cancer, 15.7% in HER-2-positive breast cancer). In patients with low baseline Ki67 or POAI-induced low Ki67 associated with good prognosis, adjuvant standard endocrine therapy and high POAI-induced Ki67 might benefit from further adjuvant treatment or trials of new therapies. The Ki67 change after 2 weeks of NET provided substantially more prognostic information for those who had high baseline Ki67.

Clinical practice is unequal to clinical trials, and every patient is unique. In our clinical practice, some patients need time to accept their disease and the subsequent treatment. Perhaps it is just a temporary choice for some ER-positive HER-2-negative breast cancer patients who refuse chemotherapy because of its side effects. The NET, Ki67, and PEPI systems are useful tools that provide useful information about screening for de-escalation treatment in low-risk patients. Especially in times of crisis, such as during the coronavirus disease 2019 (COVID-19) pandemic, it is of paramount importance for most patients to reduce or postpone visits to the hospital (56, 57). The NET, Ki67, and PEPI systems are alternative choices for ER-positive HER-2-negative breast cancer. However, 5%–20% of ER-positive HER-2-negative breast cancers have clinical progression (58). As we know, the data about axillary lymph nodes after NET remain limited; no research majored on the relationship between the Ki67 index and axillary lymph node response to NET. A previous study indicated that NETs can have equivalent clinical benefit to neoadjuvant chemotherapy in appropriately selected patients (59). According to the subtype of breast cancer, the attitudes of the patients and family members, and the information provided by clinical trials, the determination of NET should be cautious and followed up closely. For patients who demonstrate early endocrine resistance to NETs, additional adjuvant systemic therapy should consider alternative treatment approaches to reduce recurrence risk and aggression.

## Ki67 Measurement in Neoadjuvant Endocrine Therapy

Ki67 measures the proportion of proliferating cells in breast cancer. Ki67 IHC has been used for many years and is reported by pathologists as a Ki67 index in the clinic. However, Ki67 is not completely integrated in clinical decision-making because of a lack of a standardized procedure for Ki67 assessment as well as persistence of several issues of debate with regard to the Ki67 assay interpretation and the marker’s clinical utility. With the goal of establishing a uniform Ki67 evaluation system, the International Ki67 in Breast Cancer Working Group of the Breast International Group and North American Breast Cancer Group conducted a Ki67 reproducibility study. They found that tumor region selection, hot spot analysis, counting method, and subjective assessment of staining positivity resulted in interlaboratory discordance (60–62). A set of guidelines for staining, analysis, and reporting of Ki67 is recommended by the IKWG (5, 28).

The cutoff for Ki67 is still under debate. Published Ki-67 data from the IMPACT and P024 were used for the development of cutoff points for prospective validation. In the IMPACT trial, the geometric mean percentage change of Ki67 after 2 and 12 weeks of NTS was greater in the anastrozole group (76.0% and 81.6%) than in the tamoxifen group (59.5.0% and 61.9%) or the combination group (63.9% and 61.1%) (47). In P024, letrozole inhibited Ki67 to a greater extent than tamoxifen did (reduction in geometric mean Ki67 level 87% vs. 75%, respectively; *P* = 0.0009) (42, 46). The PEPI score was established in the P024 trial and validated in IMPACT trial (46, 63). It combines the residual Ki67 score, which was analyzed as the natural log interval, or per 2.7-fold increase according to the original scale of percentage values (53, 63). The Z1031 study established a Ki67 cutoff point for triage to chemotherapy after 2 weeks of AI therapy (56). If Ki67 ≤10%, the patient continued AI therapy for another 12–14 weeks and then proceeded to surgery. If the Ki67 level was >10%, the patients were offered either neoadjuvant chemotherapy or surgery. In HORGEN and CARMINA02, the cutoff of Ki67 expression is ≤10% vs. >10% (55). In the POETIC clinical trial, the cutoff was <10% vs. ≥10% (28). The change in Ki67 is of predictive value in NET (28, 38). Currently, the evaluation of Ki67 is considered important in clinical practice, especially in neoadjuvant endocrine clinical trials, and standardized and accurate evaluation under strict quality control is needed. Unless the assessment is carried out in an experienced laboratory with its own reference data and strict quality control, it is not reliable to directly apply a specific cutoff value to make decisions.

## Conclusion

Ki67 is a useful proliferation marker; its potential usefulness in predicting response and long-term outcome is explored in NET. It cannot represent or predict the regression of the primary tumor or lymph node after NET. It can be used as a predictor to evaluate the recurrence-free survival rate of patients, or its change can be used as the preoperative “window of opportunity” in NET. At present, a set of guidelines for staining, analysis, and reporting of Ki67 is recommended in breast cancer, but the uniformity among different centers needs to be improved. Standardized NET, Ki67, and PEPI systems require further standardization and subsequent clinical validation.

In clinical practice, the aim of neoadjuvant therapy is to shrink or downstage breast cancer, increase the breast conservation rate, and help to screen appropriate patients for de-escalation or escalation therapy, regardless of neoadjuvant chemotherapy or NET. For triple-negative and HER-2-positive breast cancer, neoadjuvant chemotherapy is the first choice. For ER-positive and HER-2-negative breast cancer, is NAC or NET the best choice or first choice? With large tumor burden, should NAC or NET be selected? With lymph node metastasis, should NAC or NET be selected? Ki67 may offer clues. Previous reports indicated that a higher pretreatment Ki67 was more likely to attain pCR after NAC and can be used as a predictor of NAC in luminal subtypes only (3, 4, 64). This suggests that higher pretreatment Ki67 may improve the prognostic significance of clinical response in NAC. Due to the uniformly low pCR and slow response (65, 66), NETs are not the first choice for the quick downstaging of large tumor burden. Due to the limited data on axillary management or outcomes in NET clinical trials, most patients selected for NETs have limited nodal burden. More research is needed.

## Author Contributions

All authors made substantial contributions to articles reviewed in this manuscript, were involved in the drafting and revision, and approved the final version of this manuscript.

## Funding

This work was supported by the National Natural Science Foundation of China (No. 81972791). The funders had no role in study design, data collection and analysis, decision to publish, or preparation of the article.

## Conflict of Interest

The authors declare that the research was conducted in the absence of any commercial or financial relationships that could be construed as a potential conflict of interest.

## Publisher’s Note

All claims expressed in this article are solely those of the authors and do not necessarily represent those of their affiliated organizations, or those of the publisher, the editors and the reviewers. Any product that may be evaluated in this article, or claim that may be made by its manufacturer, is not guaranteed or endorsed by the publisher.

## Footnotes

1 PEST: The amino acid sequences of ten proteins with intracellular halflives less than 2 hours contain one or more regions rich in proline (P), glutamicacid (E), serine (S), and threonine (T).
